# Parenthood is associated with lower suicide risk: a register‐based cohort study of 1.5 million Swedes

**DOI:** 10.1111/acps.13240

**Published:** 2020-10-19

**Authors:** Marina Dehara, Michael B. Wells, Hugo Sjöqvist, Kyriaki Kosidou, Christina Dalman, Alma Sörberg Wallin

**Affiliations:** ^1^ Clinical Epidemiology Division Department of Medicine Solna Karolinska Institutet Stockholm Sweden; ^2^ Department of Global Public Health Karolinska Institutet Stockholm Sweden; ^3^ Department of Women's and Children's Health Karolinska Institutet Stockholm Sweden; ^4^ Centre for Epidemiology and Community Medicine Region Stockholm Stockholm Sweden

**Keywords:** suicide, parenthood status, children, sex differences, cohort study

## Abstract

**Objective:**

To determine whether parenthood among 25‐ to 44*‐*year*‐*old*s* is associated with a lower suicide rate in men and women in Sweden, and whether this is explained by selection into parenthood.

**Methods:**

In total, 1,582,360 Swedish women and men, born between 1967 and 1985, and childless at their 25^th^ birthday, were followed from 1992 to 2011. All data originated from linkage to national Swedish registers. Cox regression models were used with time‐varying parenthood status to estimate adjusted hazard ratios and 95% confidence intervals (aHR;CI) for suicide.

**Results:**

Having one, two, three or more children was associated with 64%, 79% and 78% lower suicide rate, respectively, compared with having no children, in models with basic adjustments. When a wide range of indicators of selection into parenthood were taken into account, the suicide rate was 58% lower in parents with one child and 70% lower in parents with two or more children compared with childless individuals (aHR 0.42 [95% CI 0.36‐0.48]; 0.30 [95% CI 0.25‐0.35]; 0.30 [95% CI 0.21‐0.42]). In fathers with one, two, three or more children suicide rate was 54%, 64% and 59% lower, respectively, compared with non‐fathers whereas in mothers was 70%, 83% and 93% lower, respectively, compared with non‐mothers.

**Conclusion:**

Parenthood among 25‐to 44*‐*year*‐*old*s* is associated with a lower suicide risk in both men and women but to a larger extent among women, and particularly in parents with two or more children. Although selection into parenthood is possible, a protective effect of parenthood on suicide is likely in both men and women.

**Significant outcomes**

- Our study among 1.5 million adults in Sweden shows that parenthood, between ages 25 and 44, is associated with lower suicide risk compared with non‐parents in Sweden, with a particularly low risk in parents with two or more children (70%).
- The lower suicide risk in parents is evident in both men and women, but it is more pronounced among women.
- Selection into parenthood seems to explain a part of the association between parenthood and suicide, but the lower risk in parents still remains even after controlling for a range of indicators of selection.

**Limitations**

- The exclusion of people who had become parents by the age of 25 might impact the generalizability to parents younger than 25.
- Some residual confounding is likely due to data limitations, such that information on psychiatric disorders only captured inpatient but not outpatient data; and the indicators of selection were measured in early adulthood but are likely to change over time.

## 1 INTRODUCTION

In Sweden, more than 1500 people die each year due to suicide, where around one‐third are adults of childbearing age (aged 25‐44).^1^ During the last twenty years, the suicide rate has increased by almost 1% per year among 25‐ to 44‐year‐olds, a trend that has now been established.^1^

Suicide is a complex multi‐causal event influenced by several factors, including risk, as well as protective factors.^2^ According to the interpersonal theory of suicide (ITS),^3^ a factor that might help reduce the suicide risk is parenthood, which is defined here as 'the state of being a parent'.^4^ The ITS posits that the presence of children can decrease the feelings of loneliness, which in turn can decrease thwarted belongingness, and thus help to reduce suicidal ideation.^3^ Previous studies support the ITS by indicating that parenthood is associated with a decreased suicide risk^5^, ^6^, ^7^, ^8^, ^9^, ^10^, ^11^, ^12^, ^13^, ^14^, ^15^ and that the risk of suicide decreases gradually with an increasing number of children.^7^, ^8^, ^9^, ^10^ However, issues of statistical power^6^, ^9^ and study designs, which are mostly case‐control^13^, ^14^, ^15^ or cross‐sectional designs,^7^, ^12^ or only include parts of the population, such as married women^8^ or women with at least one child^9^, ^10^ or focused on living arrangements rather than parenthood,^5^, ^11^ limit their generalizability and conclusions.

In addition, it has not been clearly elucidated if the protective effect of parenthood on suicide risk can be explained by differences in factors that influence selection into either becoming a parent or remaining childless (hereinafter referred to as 'selection into parenthood'). For instance, physically and mentally healthy people are more likely to have children^16^ while being single,^17^ having higher education levels, employment/career planning^17^, ^18^ and living in an area with high population density^19^, ^20^ have been linked with delayed childbearing and lower fertility. Moreover, being woman, and being not employed increases the likelihood of remaining childless.^21^ These selection factors are also associated with suicide risk.^14^, ^15^ Only a few studies have accounted for these factors, including both women and men.^13^, ^14^, ^15^ Nonetheless, these studies were all based on the same Danish cohort and included suicide deaths between 1983 and 1997.^13^, ^14^, ^15^ They found that parenthood was more robustly associated with low suicide risk in women than in men. However, the aforementioned selection factors were measured close in time to the suicide and therefore also after the selection into parenthood would have occurred, which may have led to over‐adjusted estimates. Moreover, no previous study has been able to account for personality aspects, cognitive ability/intelligence quotient (IQ), physical fitness and childhood socio‐economic status. The first three individual characteristics are established predictors of mental health outcomes^22^, ^23^ and the latter of suicide^24^; all of which might influence selection into parenthood.^25^, ^26^, ^27^, ^28^ For example, men with very low IQ scores are more likely to be childless or have only one child,^25^ while extraversion (a personality trait)^26^ and having higher levels of physical fitness^27^ have been associated with higher lifetime reproductive success and fertility in men.

Further, even though men are two to three times more likely to die by suicide than women,^2^ differences between men and women in the association between parenthood and suicide have, to our knowledge, only been explored in the above‐mentioned Danish studies.^13^, ^14^, ^15^ They found that parenthood was associated with a lower suicide risk in both mothers and fathers, but the associations were considerably more robust in women than in men when the confounders were taken into account.^13^, ^14^, ^15^ However, the fathers’ role as child caretakers has changed rapidly in recent decades, where fathers have taken on more infant caring responsibilities since the mid‐1990s.^29^ In addition, Sweden stands out in promoting gender equality in parenting while Denmark has weaker policies for fathers.^30^ Thus, the association between fatherhood and suicide may differ between these contexts.

### 1.1 Aims of the study

We aimed to determine whether parenthood among 25‐ to 44‐year*‐*old*s* is associated with a lower rate of suicide in men and women in Sweden. We explored whether the associations, if any, were explained by selection into parenthood by taking into account a wide range of possible indicators of such selection such as socio‐demographic, labour market and psychiatric factors in participants, and personality aspects, IQ and physical fitness in a substantial subsample of men.

## 2 MATERIAL AND METHODS

### 2.1 Study design and population

We established a population‐based nationwide cohort from the Psychiatry Sweden (PS) database, which is a comprehensive register linkage especially designed for mental health research. The data were linked by Statistics Sweden, a governmental agency, using the unique personal identification number assigned to all Swedish residents, and was anonymized before making it available for research. The current study is based on individuals who were born in Sweden between 1967 and 1985, and were childless at baseline in order to avoid misclassification of mediators as confounders. The baseline was set to their 25^th^ birthday because some adjustment variables such as labour market factors and education could be less informative at younger ages. After exclusions, 1,582,360 individuals were kept for analysis (Figure S1). They were followed from the beginning of 1992, or the year they turned 25 until the time of death by suicide, death by other causes, emigration or end of follow‐up (end of 2011), whichever occurred first.

### 2.2 Ethics

Ethical approval for this study was obtained from the Regional Ethics Review Board in Stockholm, Sweden (decision reference numbers 2010/1185‐31/5 and 2013/1118‐32).

### 2.3 Exposure

Parenthood was defined as the number of biological and adopted children registered in Sweden during the study period from 1992 to 2011, and who did not die before the age of 18 during this period since parenthood, in terms of parental roles, may be vastly different if the child is deceased. The date of birth or adoption of all children was retrieved from the Total Population Register (TPR)^31^ and linked with the study participants through the Multi‐Generation Register.^32^ The number of children variable was categorized into no children, one child, two children, and three or more children, with a yearly update during the study period. The distribution of suicides by the number of children up to four or more children is presented in Figure S2; there were no suicides among parents with five or more children. The average age at the birth of the first and last child, respectively, during the study period was 29 and 31 in women and 30 and 32 in men.

### 2.4 Outcome

Suicide was defined from the underlying cause of death in the Cause of Death Register^33^ between 1992 and 2011. The following International Classification of Diseases (ICD) codes were used to identify suicide: certain suicides, E950‐E959 (ICD‐9) and X60‐X84 [ICD‐10]; uncertain suicides, E980‐E989 (ICD‐9) and Y10‐Y34 (ICD‐10). Uncertain suicides were included in the current study since previous studies have suggested that they were likely to be suicides.^34^, ^35^

### 2.5 Indicators of selection

Potentially confounding factors, used in the present context as indicators of selection into parenthood, were selected based on previous research that showed an association with both parenthood and suicide.^13^, ^14^, ^15^ All variables were retrieved before the start of the follow‐up (1992, or the year the participants turned 25). A detailed description of the confounders is presented in Table S1. Information on sex, country of origin, marital status at age 24 and birth year was obtained from the TPR. Population density in the participants’ residential area was retrieved at age 24 from the TPR by using Statistics Sweden’s 'Small Area Marketing Statistics' classification system. Information on unemployment at age 23 and 24, social benefit at age 23 or 24, sickness leave/disability pension at age 23 or 24 and participant’s education level at age 24 was retrieved from the Longitudinal Integration Database for Health Insurance and Labor Market Studies.^36^ Information on childhood socio‐economic position (SEP) was obtained from the Population and Housing Census.^36^ Data on participant's admissions with a psychiatric disorder and suicide attempt were obtained from the Inpatient register^37^ and defined as any admission up until the end of the year when the participant turned 24. Data on IQ, personality aspects including emotional control and social maturity, physical fitness and any psychiatric diagnoses were obtained from the Swedish Military Conscription Registry, which contains data from mandatory conscription of nearly all men at age 18‐20. These variables have been described in detail elsewhere, and they are all associated with subsequent suicidal behaviour in Swedish men.^22^, ^38^ The conscription data were linked to a subset of the male study population who were conscripted for compulsory military service in the available years, 1984‐1997. Since conscription was mandatory, this subset comprises nearly all men born 1967‐1979 (68% of the total male study population) and is therefore highly representative.

### 2.6 Statistical analyses

The associations between parenthood and suicide were estimated using Cox regression models with age as the underlying time‐scale. The number of children was modelled as a categorical, time‐varying variable with yearly updates, and hazard ratios (HR) were calculated for 1 child, 2 children, and 3 or more children, while those without children were treated as the reference group. Two models were estimated in a stepwise manner to assess possible selection effects on the associations between parenthood and suicide. Model 1 adjusted for cohort effect (categorized into four birth cohorts based on birth year: 1967‐1970, 1971‐1975, 1976‐1980, 1981‐1985), sex and country of origin since those variables in most cases remained stable during the lifespan. Model 2 included, additionally, all indicators of selection, namely population density, childhood SEP, unemployment, social benefit, sick leave/disability pension, marital status, education, and history of psychiatric disorder and suicide attempt. A possible interaction between the number of children and sex was further investigated using the nested likelihood ratio (LR) test, and we then repeated the analysis stratified by sex. Censoring was made at the time of suicide death, death by other causes, emigration or at the end of the study, whichever comes first. We examined the HRs of the number of children graphically over time and found them to be approximately proportional. The analysis was repeated in the subset of men with conscription data for additional control of potential selection into parenthood. Two sensitivity analyses were conducted to examine the robustness of the results; 1) by excluding adoptive parents from the exposure (retaining only biological parents), 2) by excluding uncertain suicides from the outcome (retaining only ICD codes E950‐9 [ICD‐9] and X60‐X84 [ICD‐10]). All statistical analyses were conducted in Stata software, version 15.0. Most of the data structuring was done using SAS software, version 9.4.

## 3 RESULTS

### 3.1 Characteristics of the study population

Table 1 shows the characteristics of study participants born between 1967 and 1985 who did and did not die from suicide, between 1992 and 2011. A total of 2,453 deaths from suicide occurred among 1,582,360 study participants during a 1‐ to 19‐year follow‐up period. Men accounted for over three‐quarters of all suicides. Living in an area with low population density was more common among those who had died from suicide, with this pattern holding true for men but the opposite was true for women. All other conditions including low childhood SEP, having only compulsory education, unemployment, receiving social benefits, or having a history of psychiatric disorder or suicide attempt were more common in those who later died from suicide, in both women and men. Moreover, being born between 1967 and 1970 was more common among those who had died from suicide, but those participants had a longer follow‐up period; the annual suicide rate was more similar between the four cohorts (annual rate per 100,000: 16.0, 14.4, 16.0 and 19.7, respectively). The distribution of the participants' characteristics across the number of children and the characteristics measured at conscription in fathers and non‐fathers are presented in Tables S2 and S3, respectively.

**Table 1:** Socio‐demographic characteristics, history of psychiatric disorder and suicide attempt of study participants who did and did not die from suicide, birth year 1967‐1985 (N = 1,582,360)

	Total N = 1,582,360		Men n = 871,598		Women n = 710,762	
	Non‐suicides N = 1,579,907	Suicides N = 2,453	Non‐suicides n = 869,719	Suicides n = 1,879	Non‐suicides n = 710,188	Suicides n = 574
Sex male	869,719 (55.1)	1,879 (76.6)				
Cohort (1967‐1970)	341,238 (21.6)	916 (37.3)	193,197 (22.2)	698 (37.2)	148,041 (20.9)	218 (38.0)
Non‐Swedish‐born	76,814 (5.0)	197 (8.0)	41,758 (4.8)	141 (7.5)	35,056 (4.9)	56 (9.8)
Low Population density^a^	427,165 (27.0)	737 (30.0)	255,814 (29.4)	612 (32.6)	171,351 (24.1)	125 (21.8)
Low Childhood SEP	588,151 (37.2)	1,006 (41.0)	330,986 (38.1)	782 (41.6)	257,165 (36.2)	224 (39.0)
Unemployed^b^	233,407 (14.8)	497 (20.3)	120,388 (13.8)	398 (21.2)	113,019 (15.9)	99 (17.3)
Social benefit^b^	157,950 (10.0)	845 (34.5)	91,310 (10.5)	643 (34.2)	66,640 (9.4)	202 (35.2)
Sick leave/disability pension^b^	276,995 (17.5)	945 (38.5)	146,057 (16.8)	678 (36.1)	130,938 (18.4)	267 (46.5)
Only compulsory education^a^	147,357 (9.3)	720 (29.4)	97,961 (11.3)	563 (30.0)	49,396 (7.0)	157 (27.4)
Unmarried/divorced^a^	1,539,276 (97.4)	2,423 (98.8)	855,602 (98.4)	1,866 (99.3)	683,674 (96.3)	557 (97.0)
History of Psychiatric disorder^a^	62,453 (4.0)	705 (28.7)	33,439 (3.8)	477 (25.4)	29,014 (4.1)	228 (39.7)
History of Suicide attempt^a^	19,091 (1.2)	345 (14.1)	7,437 (0.9)	193 (10.3)	11,654 (1.6)	152 (26.5)

Abbreviation: SEP, socio‐economic position.

^a^ Measured at age 24.

^b^ Measured at age 23 and 24.

Data are given as number (percentages); all variables are presented as binary only for descriptive purposes, but the categories described in Table S1 were used in the Cox regression models.

### 3.2 Association between parenthood and risk of suicide

Table 2 shows the associations between the number of children and suicide in the total study population. In Model 1, having one, two, three or more children was associated with 64%, 79% and 78% lower suicide rate, respectively, compared with having no children. The estimates were attenuated in Model 2, but a considerably lower risk of suicide was still associated with having children, compared with not having children. Even after the adjustments in Model 2, having one child was associated with a 58% lower risk of suicide, and having two children and having three or more children with a 70% lower risk, compared with having no children.

**Table 2:** Hazard ratios (HR) and 95% confidence intervals (CI) for the risk of death by suicide by number of children in the study population of Swedish individuals born 1967‐1985 (N = 1,582,360)

Number of children	Model 1^a^	Model 2^b^
	HR [95% CI]	HR [95% CI]
No children^c^	1.00	1.00
1 child	0.36 [0.32‐0.41]*	0.42 [0.36‐0.48]*
2 children	0.21 [0.18‐0.25]*	0.30 [0.25‐0.35]*
3 or more children	0.22 [0.15‐0.30]*	0.30 [0.21‐0.42]*

^a^ Model 1: adjusted for cohort effect; sex; country of origin.

^b^ Model 2: adjusted for model 1 + childhood socio‐economic position; unemployment at age 23 and 24; social benefit and sick leave/disability pension at age 23 or 24; marital status, population density, education, history of psychiatric disorder and history of suicide attempt at age 24.

^c^ Reference.

* P < 0.001.

### 3.3 Sex‐stratified analyses

The LR test indicated an interaction between the number of children and the participants’ sex in predicting suicide risk (*P* < 0.001), and therefore, further Cox regression models stratified by sex were conducted.

Table 3 shows the associations between the number of children and suicide for men. In Model 1, fathers with one, two, three or more children had a 59%, 74% and 69% lower risk of suicide, respectively, compared with non‐fathers. The estimates were attenuated in Model 2, but a considerably lower risk of suicide was still observed in the fathers. Specifically, in Model 2, fathers with one, two, three or more children had a 54%, 64% and 59% lower risk of suicide, respectively, compared with non‐fathers. The lowest risk was observed in fathers with two or more children; for fathers with two versus fathers with three or more children, the point estimates were fairly similar and the confidence intervals were overlapping. In the subset of men with conscription data, the estimates for Models 1 and 2 were only slightly lower than the estimates in the larger sample of men, suggesting that they were representative of the larger sample (Table S4). Adding the variables measured at conscription at age 18 to Model 2 attenuated the associations minimally. For example, men with two children had a HR of 0.31 (95% CI 0.24‐0.39) in Model 2, while with additional adjustment for the conscription variables, the HR was 0.32 (95% CI 0.26‐0.41).

**Table 3:** Hazard ratios (HR) and 95% confidence intervals (CI) for the risk of death by suicide by number of children for men, birth year 1967‐1985 (n = 871,598)

Number of children	Model 1^a^	Model 2^b^
	HR [95% CI]	HR [95% CI]
No children^c^	1.00	1.00
1 child	0.41 [0.35‐0.48]*	0.46 [0.40‐0.54]*
2 children	0.26 [0.22‐0.32]*	0.36 [0.30‐0.43]*
3 or more children	0.31 [0.22‐0.44]*	0.41 [0.29‐0.59]*

^a^ Model 1: adjusted for cohort effect; country of origin.

^b^ Model 2: adjusted for model 1 + childhood socio‐economic position; unemployment at age 23 and 24; social benefit and sick leave/disability pension at age 23 or 24; marital status, population density, education, history of psychiatric disorder and history of suicide attempt at age 24.

^c^ Reference.

* *P* < 0.001.

Table 4 shows the associations between the number of children and suicide for women. In Model 1, mothers with one, two, three or more children had a 75%, 89% and 96% lower risk of suicide, respectively, compared with non‐mothers. The estimates were attenuated in Model 2, but a considerably lower risk of suicide was still observed in the mothers. Specifically, in Model 2, mothers with one, two, three or more children had a 70%, 83% and 93% lower risk of suicide, respectively, compared with non‐mothers. Mothers with two or more children had the lowest risks, with no distinguishable difference in suicide risk between mothers with two and mothers with three or more children since the confidence intervals were overlapping.

**Table 4:** Hazard ratios (HR) and 95% confidence intervals (CI) for the risk of death by suicide by number of children for women, birth year 1967‐1985 (n = 710,762)

Number of children	Model 1^a^	Model 2^b^
	HR [95% CI]	HR [95% CI]
No children^c^	1.00	1.00
1 child	0.25 [0.19‐0.33]*	0.30 [0.23‐0.40]*
2 children	0.11 [0.07‐0.15]*	0.17 [0.12‐0.24]*
3 or more children	0.04 [0.01‐0.14]*	0.07 [0.02‐0.22]*

^a^ Model 1: adjusted for cohort effect; country of origin.

^b^ Model 2: adjusted for model 1 + childhood socio‐economic position; unemployment at age 23 and 24; social benefit and sick leave/disability pension at age 23 or 24; marital status, population density, education, history of psychiatric disorder and history of suicide attempt at age 24.

^c^ Reference.

* *P* < 0.001.

### 3.4 Sensitivity analyses

To examine the robustness of the results, two sensitivity analyses were performed. In the first one, parents with adopted children were excluded (Table S5). In the second, uncertain suicides (retaining only ICD codes E950‐9 [ICD‐9] and X60‐X84 [ICD‐10]) were excluded from the outcome and censoring them as deaths from other causes (Table S6). Both of these analyses yielded very similar estimates compared with the main analyses. Thus, the observed associations were not heavily affected by either a potential misclassification of suicide or the subgroup of parents with adopted children.

## 4 DISCUSSION

Swedish people between 25 and 44 years of age had a substantially lower risk for suicide if they had children, even after adjusting for a wide range of individual background factors, such as socio‐demographic, labour market and psychiatric factors in participants; and personality aspects, IQ and physical fitness in a subsample of male participants. When data were stratified by sex, the association was evident for both women and men, but it was stronger among women. Mothers and fathers with only one child had a considerably lower suicide risk compared with non‐parents, but the lowest risks were observed in those with two or more children.

### 4.1 Comparison with previous studies

In line with previous findings, parenthood was associated with reduced suicide risk in women.^5^, ^6^, ^7^, ^8^, ^9^, ^10^, ^11^, ^13^ For example, in a large Danish population‐based study, having one child was associated with a 28% lower risk compared with women without children.^13^ This study is also, to our knowledge, the only one which focuses on parenthood and suicide in men as well, and in unadjusted models, fathers had a similar reduction in suicide risk as mothers.^13^ The weaker association than in the present study might be explained by the larger age span, 18‐64 years. Additionally, a nested case‐control design and logistic regression were used in that study, which might yield different estimates than the cohort design and Cox regressions that we used in the present study. In a comparison between Cox and logistic regression, Cox regression was found to yield less biased estimates for population‐based data.^39^

Our findings of a lower suicide risk in parents even in the adjusted models stand in contrast with the Danish study.^13^ In that study, the suicide risk for parents with one, two, or three to four children was substantially reduced and not statistically significant in the total sample after adjusting for covariates, with the same results for men as for the total sample in sex‐stratified analyses. For women, however, the reduced suicide risk of having children remained statistically significant for mothers with three to four children, with a 16% lower suicide risk compared with non‐mothers after adjusting for covariates. However, part of the explanatory effect from these covariates might be mediation instead of selection or confounding since conditions such as marital status, labour market status and income were measured the year before the year of suicide and might have been affected by parental status. Thus, it is unclear whether appropriate temporality of confounding was ensured.

Other studies on women have found indications that the risk of suicide is lower with an increasing number of children,^7^, ^8^, ^9^, ^10^ but they did not adjust for a similar set of explanatory factors. Also, because of differences in design, such as including only women who had at least one child^9^, ^10^ or presenting absolute rates instead of hazard ratios,^7^ direct comparisons with our findings are limited.

### 4.2 Interpretation of findings

The ITS proposes that having children can reduce the feeling of loneliness, which in turn reduces thwarted belongingness, and hence help to reduce a person's suicidal ideation.^3^ Similarly, the presence of children may induce a greater purpose and direction in a parent’s life, which includes a sense of obligation, responsibility and care‐taking, and a feeling of belongingness.^5^, ^40^, ^41^ An alternative explanation is that children might be an important source of social and emotional support for parents that may buffer poor health and early mortality.^42^, ^43^ Moreover, having children may provide parents with new opportunities to further broaden their social connectedness or social support network by meeting other parents through their children, as well as by providing access to acquaintances, friends and to extended family and non‐family members, such as neighbours,^44^, ^45^ and therefore be less lonely. Regarding our finding of lower risks in parents with at least two children, having more than one child might increase the sense of belongingness and support, and also increase social connectedness since additional children are likely to increase the contact with other adults in the children’s environment. Moreover, since couples who separate after having one child may be less likely to have additional children, relationship stability could partly account for the lower suicide risk in parents with more than one child.

However, as the association was attenuated when taking possible selection factors into account, selection into parenthood explains some of the association. For example, it is known that individuals that are physically and mentally healthy are more likely to become parents,^16^ while housing and financial uncertainty, employment and career factors, intellect, sociability and relationship status may impact on opportunities and the decision of having children.^18^, ^46^, ^47^ In line with these theories, we did expect that selection would explain a substantial part of the association between parental status and suicide. However, although the wide range of possible indicators of selection had an attenuating effect on the associations, the association was still substantial in men and women alike. Thus, even though some residual selection is possible, we argue that a protective effect from parenthood is likely for both men and women.

In Sweden, family‐friendly policies, such as parental leave, encourage both parents to be involved in childrearing.^48^ For instance, fathers in Sweden are taking an increasing amount of parental leave^49^ and their amount of parental leave days taken has positive effects on fathers’ participation in childcare and relations with their children.^50^ These family‐friendly policies may explain our findings that both fathers and mothers had a lower risk of suicide, as parents are not just having children, but also spend an increasing amount of time with their young children. However, mothers had a larger decrease in suicide risk compared with fathers. This may be due to mothers still being more likely to be the primary caregiver, and thus, spend more time in childrearing roles.^51^

### 4.3 Strengths and limitations

The main strength of this cohort study was the data completeness with minimal selection bias or attrition, and a wide range of adjustment factors associated with parenthood and suicide in the PS database. The prospective and objective data collection in the national registers made the risk of recall bias for our exposure, outcome and selection indicators negligible.

Since the data are gathered from the total Swedish population, the generalizability of the findings is high in Sweden. Similarly, since our findings of reduced suicide risk with having children are generally in line with previous studies,^5^, ^6^, ^7^, ^8^, ^9^, ^10^, ^11^, ^12^, ^13^ they may also partly transfer to other high‐income countries. However, further research in other countries is necessary to better understand the generalization of these findings.

The lower limit of age 25 was chosen since the indicators of selection, such as unemployment, social and sick leave benefits, and educational attainment, available in our data, are arguably better measured at age of 23‐24 than at earlier age. At the age of 24, people have often reached their highest education level and are recently established in the labour market, and these variables are therefore not as relevant at a younger age. Moreover, we had no information on the number of days with unemployment, social and sick leave benefits but based our binary variables on income data. However, given the small groups of people who received these benefits, they are quite strong risk markers even as binary variables.

Socio‐economic, marital and mental health status may be confounders in the association between parenthood and suicide.^14^, ^15^ However, these variables can act both as confounders and mediators if they occur after parenthood. Thus, to ensure appropriate temporality of exposure, confounders and outcome, we started the observation time at age 25 years, with only childless individuals, so that mediators are not adjusted in the multivariable models. However, some residual confounding is likely due to this more conservative approach regarding mediators. For instance, some confounders, such as unemployment and marital status, were measured at age of 23‐24 but are likely to change over time. The average age of getting married in Sweden is 33 in women and 36 in men,^48^ which is even higher than the average age of first‐time parents, at 29 in women and 31 in men.^51^ Moreover, the results from the present study might not be generalized to parents younger than 25. Becoming a parent at a young age may imply stressors that act opposite to a reduced suicide risk with having a child, and thus, we would expect our estimates to be slightly weaker if we had included them. Moreover, in our study, the oldest age was 44; thus, our results may not be generalizable without caution to older age groups.

Moreover, some residual confounding is likely due to data limitations, such that information on psychiatric disorders and suicide attempts was fully available only from the inpatient register, and people treated only in outpatient care were therefore not captured, which might have attenuated the associations additionally. In addition, no data on cohabitation were available which is common in Sweden, especially in young people.

In the overall study population, there are more men than women (871,598 men compared with 710,762 women) since more women than men had their first child before age 25. Thus, the generalizability is slightly more limited for women.

### 4.4 Public health implications and future research

Given the urgent need for more knowledge about suicide and suicide prevention, our findings of low suicide risk with having children are highly relevant for professions in suicidological work and the public interest because they may generate new views on preventive strategies. For instance, since reduced loneliness may be a mechanism that reduces suicide risk in parents,^3^ interventions to help parents build a healthy close relationship with their children could help not only the child to a healthier life but also the parents. Moreover, clinicians should be aware that people without children may be at increased risk of suicide and consider parenthood when assessing patients.

The generous parental leave policies in Sweden and their aim for a more gender‐equal division of child care‐taking^52^ may have contributed to the low suicide risk for both men and women with children since during the study period (1992‐2011) two of the biggest parental leave policy changes occurred. In 1995, the 30 days reserved for fathers (non‐transferable leave days to the mother) was introduced to encourage fathers to take more parental leave, which was expanded to 60 days in 2002; this is referred to as 'daddy quota'.^52^ Moreover, in 2002 the total number of days for the parents was prolonged from 450 to 480 days.^52^ Although speculative, we argue that the father‐child relationship may be especially important in this context as men are two to three times more likely than women to die by suicide^2^ and because they are less likely to be the child’s primary caregiver.^51^

Future studies should investigate the potential mechanisms in the association between parenthood and suicide, such as social connectedness and caring.^3^ If such factors are found to be important, interventions to support parents in these aspects, and also to increase these factors in childless people, might be effective parts of suicide prevention. Moreover, since parental leave allows for more care‐taking and responsibility of the child, it is possible that parental leave usage is an effect modifier in the association. Similarly, residing with a child and the age of the child can be possible effect modifiers as well. For example, findings from the United Kingdom showed that females who died from suicide were reported to have a child residing at home to a lesser extent than the general female adult population.^6^ Moreover, findings from Denmark showed that having a minor child (<18 years old) who is likely to still live at home was associated with a reduced risk of suicide compared with not having a minor child.^13^ Thus, future studies should further explore those two potential effect modifiers.

Furthermore, little is known about how parenthood compares with other close relationships regarding suicide risk. For instance, taking part in work and social activities and friendships might tap into similar mechanisms as suggested by the ITS, including social connectedness and caring. Thus, future studies should explore the wider social network in association with suicide.

In sum, we found that parenthood was associated with a lower risk of suicide. The association was evident in both men and women, but was stronger among women. Selection into parenthood might still explain some of the association, but a protective effect of parenthood is likely in both men and women.

## 6 CONFLICT OF INTEREST

None declared.

7

### PEER REVIEW

The peer review history for this article is available at https://publons.com/publon/10.1111/acps.13240.

## Supporting information


**Figure S1‐S2**

## Data Availability

Data cannot be shared publicly under the terms and conditions of ethical approval for Psychiatry Sweden. This research had ethical approval obtained by the Stockholm Regional Ethical Review Board (decision reference numbers 2010/1185‐31/5 and 2013/1118‐32). Please contact the Stockholm Regional Ethical Review Board (https://etikprovningsmyndigheten.se/) for information about access to Swedish register data for researchers who meet the criteria for access to confidential data.
