# Supplementary material for: Parenthood is associated with lower suicide risk: a register‐based cohort study of 1.5 million Swedes
Source: Acta Psychiatr Scand. 2020 Oct 19;143(3):206–15. doi: 10.1111/acps.13240 (PMC7983926; doi:10.1111/acps.13240)
Supplement: Supplementary file 1 — Figure S1‐S2 [file ACPS-143-206-s001.docx]

**Supplementary Material**

Final sample for analysis

N=1,582,360

Birth cohort 1967-1985

N=1,918,322

Unknown parental status

n=2,352

Parents who had a first child before the age of 25

n=320,114

Total missing data on the confounders

n=8,835

-Sickness/disability (n=50)

-Unemployment (n=58)

-Social benefit (n=58)

-Education (n=8,793)

Missing date on suicide cases

n=89

Parents with a child who died

<18 years old

n=4,572

Supplementary Figure 1. Flow-chart of the study population

Supplementary Figure 2. The number of deaths by suicides in men and women, by the number of children.

| Supplementary Table 1. Description of the indicators of selection/confounders | | | |
| --- | --- | --- | --- |
| **Variable** | **Type** | **Source** | **Comment** |
| Sex | Binary: Women; Men | TPR† |  |
| Country of origin | Categorical: Sweden; Other Nordic; Other countries | TPR† |  |
| Marital status | Categorical: Unmarried; Married; Divorced | TPR† | Retrieved at age 24. No information on cohabitation was available |
| Birth year | Categorical: 1967-1970; 1971-1975; 1976-1980; 1981-1985 | TPR† | Categorized into birth cohorts to avoid multicollinearity. In the adjusted models, this variable is mentioned as “cohort effect”. By adjusting for the year of birth we aimed to account for any generational differences in our study |
| Population density | Categorical: deciles (1-10). 1=lowest and10=highest | Statistics Sweden’s SAMS‡ classification system and the TPR† | Retrieved at age 24. The SAMS‡ is a classification of demographically homogeneous geographical areas into 9,208 neighbourhoods. Population density was calculated by dividing the number of residents by the geographical land area. Participants with no registered residence were placed in a separate category |
| Unemployed | Binary: Yes; No | LISA§. Complete coverage since 1990 | Defined according to unemployment benefits. Unemployment was categorized as “yes” if the participants had any benefits in both years, when the participant turned 23 and 24 years old. Information on unemployment benefits available as the annual amount of monetary benefit (no data on days with benefits was available) |
| (continued) | | | |

| Supplementary Table 1. (continued) | | | |
| --- | --- | --- | --- |
| **Variable** | **Type** | **Source** | **Comment** |
| Social benefit | Binary: Yes; No | LISA§ | Social benefit was categorized as “yes” if the participants had any benefits in either year, when the participant turned 23 and 24 years old, respectively. Information on social benefits available as the annual amount of monetary benefit (no data on days with benefits was available) |
| Sick leave/ disability pension | Categorical: No/little sick leave (≤2 weeks); Short-term sick leave (>2 weeks, but less than the long-term category); Long-term sick leave or disability pension. | LISA§ | Defined according to sick leave benefits. The participants were classified as receiving sick leave benefits if they had any benefits in either year, when the participant turned 23 and 24 years old, respectively. Long-term sick leave defined as receiving sick leave benefits corresponding to one “price base amount” to accommodate changes in the general price level over the two years, when the participant turned 23 and 24 years old. Information on sick leave benefits available as the annual amount of monetary benefit (no data on days with benefits was available). |
| Education | Categorical: Primary education (<9 years); Primary and lower secondary education (9 years); Upper secondary education, (2-4 years); Short post-secondary education (<2 years); Longer post-secondary education (≥2 years) | LISA§ | Retrieved at age 24 as the highest level of attained education |
| Childhood SEP¶ | Categorical: Unskilled workers; Skilled workers; Assistant non-manual employees; Non-manual employees at an intermediate level; Non-manual employees at a high level; Self-employed; Farmer; No occupation reported | The Population and Housing Census | Childhood SEP was based on self-reported occupation of the participant’s father in the census years 1980, 1985 or 1990 when the participants were 5-13 years old (mother’s occupation was used if there was no data available for the father) |
| (continued) | | | |

| Supplementary Table 1. (continued) | | | |
| --- | --- | --- | --- |
| **Variable** | **Type** | **Source** | **Comment** |
| History of Psychiatric disorder | Binary: Yes; No | IPR††. Information on psychiatric inpatient care since 1973, with relatively complete coverage and 85-95% validity of all diagnoses | ICD codes 295-315 (ICD-8), 295-319 (ICD-9) and F00-F99 (ICD-10). Data was retrieved up to the end of the year when the participant turned 24. |
| History of suicide attempt | Binary: Yes; No | IPR†† | ICD codes E950-9 (ICD-9) and X60–X84 (ICD-10); E980-9 (ICD-9) and Y10–Y34 (ICD-10). Data was retrieved up to the end of the year when the participant turned 24 |
| IQ‡‡ | Categorical: high (stanine 7-9); medium (stanine 3-6); low (stanine 1-3) | SMCR§§. It contains data from mandatory conscription of nearly all men at age 18-20 (only 2-3% were exempted, mostly due to severe disability or congenital disorders). |  |
| Emotional control | Categorical: five-point scale, 1=lowest and 5=highest | SMCR§§ |  |
| Social maturity | Categorical: five-point scale, 1=lowest and 5=highest | SMCR§§ |  |
| Psychiatric diagnoses | Binary: any; none | SMCR§§ |  |
| Physical fitness | Continuous | SMCR§§ |  |
| †TPR, The Total Population Register; ‡SAMS, Small Area Marketing Statistics; §LISA, The Longitudinal Integration Database for Health Insurance and Labor Market Studies; ¶SEP, Socio-economic position; ††IPR, The Inpatient register; ‡‡IQ, cognitive ability/intelligence quotient; §§SMCR, The Swedish Military Conscription Registry | | | |

| Supplementary Table 2. Socio-demographic characteristics, history of psychiatric disorder and suicide attempt of study participants born between 1967 and 1985 across number of children (N=1,582,360) | | | | |
| --- | --- | --- | --- | --- |
|  | **Number of children** | | | |
|  | **no children**  n=711,007 | **1 child**  n=296,795 | **2 children**  n=454,123 | **≥ 3 children**  n=120,435 |
| **Sex** |  |  |  |  |
| Men | 428,983 (60.3) | 155,905 (52.5) | 224,566 (49.5) | 62,144 (51.6) |
| Women | 282,024 (39.7) | 140,890 (47.5) | 229,557 (50.5) | 58,291 (48.4) |
| **Country of origin** |  |  |  |  |
| Sweden | 671,264 (94.4) | 282,780 (95.3) | 436,870 (96.2) | 114,435 (95.0) |
| Other Nordic | 4,797 (0.7) | 2,180 (0.7) | 3,490 (0.8) | 1,089 (0.9) |
| Other Countries | 34,946 (4.9) | 11,835 (4.0) | 13,763 (3.0) | 4,911 (4.1) |
| **Birth year** |  |  |  |  |
| 1967-1970 | 88,761 (12.5) | 56,329 (19.0) | 145,580 (32.1) | 51,484 (42.7) |
| 1971-1975 | 130,536 (18.4) | 80,839 (27.2) | 182,386 (40.1) | 52,484 (43.6) |
| 1976-1980 | 183,070 (25.7) | 96,385 (32.5) | 107,108 (23.6) | 15,622 (13.0) |
| 1981-1985 | 308,640 (43.4) | 63,242 (21.3) | 19,049 (4.2) | 845 (0.7) |
| **Population density**† |  |  |  |  |
| Decile 1 (lowest) | 61,705 (8.7) | 26,239 (8.8) | 40,342 (8.9) | 12,437 (10.3) |
| Decile 2 | 60,906 (8.6) | 26,255 (8.9) | 41,669 (9.2) | 11,196 (9.3) |
| Decile 3 | 64,815 (9.1) | 27,487 (9.2) | 43,280 (9.5) | 11,571 (9.6) |
| Decile 4 | 66,793 (9.4) | 28,298 (9.5) | 45,962 (10.1) | 11,954 (9.9) |
| Decile 5 | 71,874 (10.1) | 29,428 (9.9) | 46,897 (10.3) | 12,211 (10.1) |
| Decile 6 | 72,619 (10.2) | 30,765 (10.4) | 47,035 (10.4) | 12,160 (10.1) |
| Decile 7 | 75,826 (10.7) | 31,402 (10.6) | 46,697 (10.3) | 12,119 (10.1) |
| Decile 8 | 74,456 (10.5) | 32,190 (10.8) | 47,853 (10.5) | 12,156 (10.1) |
| Decile 9 | 78,406 (11.0) | 31,710 (10.7) | 46,358 (10.2) | 11,835 (9.8) |
| Decile 10 (highest) | 82,219 (11.5) | 32,551 (11.0) | 47,447 (10.5) | 12,572 (10.5) |
| Missing | 1,388 (0.2) | 470 (0.2) | 583 (0.1) | 224 (0.2) |
| **Childhood SEP**‡ |  |  |  |  |
| Unskilled worker | 139,016 (19.5) | 59,855 (20.2) | 84,344 (18.6) | 21,015 (17.5) |
| Skilled worker | 125,534 (17.7) | 55,655 (18.8) | 83,930 (18.5) | 19,808 (16.4) |
| Assistant non-manual employees | 70,557 (9.9) | 26,560 (8.9) | 33,808 (7.4) | 10,492 (8.7) |
| Non-manual employees at intermediate level | 43,616 (6.1) | 20,117 (6.8) | 33,707 (7.4) | 9,182 (7.6) |
| Non-manual employees at higher level | 16,910 (2.4) | 7,183 (2.4) | 13,240 (2.9) | 4,451 (3.7) |
| Self-employed | 113,576 (16.0) | 41,920 (14.1) | 66,894 (14.7) | 20,330 (16.9) |
| Farmer | 71,247 (10.0) | 30,899 (10.4) | 48,013 (10.6) | 11,512 (9.6) |
| No occupation reported | 130,551 (18.4) | 54,606 (18.4) | 90,187 (19.9) | 23,645 (19.6) |
| (continued) | | | | |

| Supplementary Table 2. (continued) | | | | |
| --- | --- | --- | --- | --- |
|  | **Number of children** | | | |
|  | **no children**  n=711,007 | **1 child**  n=296,795 | **2 children**  n=454,123 | **≥ 3 children**  n=120,435 |
| **Sick leave/disability pension**§ |  |  |  |  |
| No/little sick leave | 604,958 (85.1) | 244,436 (82.4) | 362,204 (79.8) | 92,822 (77.1) |
| Short-term sick leave | 71,793 (10.1) | 43,352 (14.6) | 82,157 (18.1) | 24,858 (20.6) |
| Long-term leave or disability pension | 34,256 (4.8) | 9,007 (3.0) | 9,762 (2.1) | 2,755 (2.3) |
| **Marital status**† |  |  |  |  |
| Unmarried | 700,789 (98.6) | 287,851 (97.0) | 436,561 (96.1) | 111,878 (92.9) |
| Married | 8,357 (1.2) | 7,895 (2.7) | 16,398 (3.6) | 8,039 (6.7) |
| Divorced | 1,861 (0.2) | 1,049 (0.3) | 1,164 (0.3) | 518 (0.4) |
| **Unemployed**§ |  |  |  |  |
| Yes | 87,843 (12.4) | 47,448 (16.0) | 77,568 (17.1) | 21,045 (17.5) |
| No | 623,164 (87.6) | 249,347 (84.0) | 376,555 (82.9) | 99,390 (82.5) |
| **Social benefit**§ |  |  |  |  |
| Yes | 82,468 (11.6) | 31,472 (10.6) | 33,976 (7.5) | 10,879 (9.0) |
| No | 628,539 (88.4) | 265,323 (89.4) | 420,147 (92.5) | 109,556 (91.0) |
| **Education**† |  |  |  |  |
| Primary education (<9 years) | 2,043 (0.3) | 453 (0.2) | 405 (0.1) | 314 (0.3) |
| Primary and lower secondary education (9 years) | 77,422 (10.9) | 28,599 (9.6) | 29,868 (6.6) | 8,973 (7.4) |
| Upper secondary education, (2–4 years) | 372,435 (52.4) | 167,807 (56.5) | 262,933 (57.9) | 66,582 (55.3) |
| Short post-secondary education (<2 years) | 106,419 (15.0) | 42,118 (14.2) | 83,775 (18.4) | 25,785 (21.4) |
| Longer post-secondary education (≥2 years) | 152,688 (21.4) | 57,818 (19.5) | 77,142 (17.0) | 18,781 (15.6) |
| **History of Psychiatric disorder**† |  |  |  |  |
| Yes | 40,911 (5.7) | 10,815 (3.6) | 8,937 (2.0) | 2,495 (2.1) |
| No | 670,096 (94.3) | 285,980 (96.4) | 445,186 (98.0) | 117,940 (97.9) |
| **History of Suicide attempt**† |  |  |  |  |
| Yes | 11,068 (1.6) | 3,827 (1.3) | 3,508 (0.8) | 1,033 (0.9) |
| No | 699,939 (98.4) | 292,968 (98.7) | 450,615 (99.2) | 119,402 (99.1) |
| †Measured at age 24; ‡SEP, Socio-economic position; §Measured at age 23 and 24; Data are given as number (percentages) | | | | |

| Supplementary Table 3. The distribution of characteristics measured at conscription, age 18-20, in the total subsample of men and in men who did not have children during the study period. In comparisons between men with no children and men with at least one child | | | | |
| --- | --- | --- | --- | --- |
|  | **Total** | | **Men with no children** | |
| Measures at conscription | N | (%/mean) | N | (%/mean) |
| **IQ**† |  |  |  |  |
| High (stanine 7-9) | 110424 | (27) | 35703 | (26) |
| Medium (stanine 3-6) | 229335 | (55) | 71531 | (53) |
| Low (stanine 1-3) | 73521 | (18) | 28073 | (21) |
| **Social maturity** |  |  |  |  |
| 5 (highest) | 10508 | (3) | 2481 | (2) |
| 4 | 116109 | (28) | 29864 | (22) |
| 3 | 225253 | (55) | 73866 | (55) |
| 2 | 55919 | (14) | 26052 | (19) |
| 1 (lowest) | 5491 | (1) | 3044 | (2) |
| **Emotional control** |  |  |  |  |
| 5 (highest) | 6499 | (2) | 1512 | (1) |
| 4 | 83241 | (20) | 20662 | (15) |
| 3 | 251697 | (61) | 80607 | (60) |
| 2 | 64880 | (16) | 28995 | (21) |
| 1 (lowest) | 6963 | (2) | 3531 | (3) |
| **Psychiatric diagnosis,** any | 10230 | (2) | 4213 | (3) |
| **Physical fitness**, in maximum working capacity (Wmax), mean | 413280 | (305) | 135307 | (296) |
| †IQ, cognitive ability/intelligence quotient; The p-values are <.001 for all variables | | | | |

| Supplementary Table 4. Hazard ratios (HR) and 95% Confidence Intervals (CI) for the risk of death by suicide in the subsample of men with conscription data, birth year 1967-1985 (n=413,280) | | | |
| --- | --- | --- | --- |
|  | **Model 1†** | **Model 2‡** | **Model 3§** |
| **Number of children** | **HR [95% CI]** | **HR [95% CI]** | **HR [95% CI]** |
| no children¶ | 1.00 | 1.00 | 1.00 |
| 1 child | 0.39 [0.32-0.47]* | 0.42 [0.35-0.52]* | 0.43 [0.36-0.53]* |
| 2 children | 0.24 [0.19-0.31]* | 0.31 [0.24-0.39]* | 0.32 [0.26-0.41]* |
| 3 or more children | 0.27 [0.17-0.42]* | 0.34 [0.21-0.53]* | 0.35 [0.22-0.56]* |
| †Model 1: adjusted for cohort effect; country of origin  ‡Model 2: adjusted for model 1 + childhood socio-economic position; unemployment at age 23 and 24; social benefit and sick leave/disability pension at age 23 or 24; marital status, population density, education, history of psychiatric disorder and history of suicide attempt at age 24  §Model 3: adjusted for model 2 + social maturity, emotional control, IQ, physical fitness, psychiatric diagnosis at age 18-20  ¶Reference  *P<0.001 | | | |

| Supplementary Table 5. Hazard ratios (HR) and 95% Confidence Intervals (CI) for the risk of death by suicide by number of biological children in the total population, birth year 1967-1985 (N=1,576,825) | |
| --- | --- |
|  | **Adjusted Analysis†** |
| **Number of children** | **HR [95% CI]** |
| no children‡ | 1.00 |
| 1 child | 0.41 [0.36-0.47]* |
| 2 children | 0.29 [0.25-0.35]* |
| 3 or more children | 0.30 [0.21-0.42]* |
| Total |  |
| †Adjusted for cohort effect; sex; country of origin; childhood socio-economic position; unemployment at age 23 and 24; social benefit and sick leave/disability pension at age 23 or 24; marital status, population density, education, history of psychiatric disorder and history of suicide attempt at age 24  ‡Reference  *P<0.001 | |

| Supplementary Table 6. Hazard ratios (HR) and 95% Confidence Intervals (CI) for the risk of death by suicide (only certain suicides) by number of children in the total population, birth year 1967-1985 (N=1,581,832) | |
| --- | --- |
|  | **Adjusted Analysis†** |
| **Number of children** | **HR [95% CI]** |
| no children‡ | 1.00 |
| 1 child | 0.42 [0.36-0.49]* |
| 2 children | 0.32 [0.27-0.38]* |
| 3 or more children | 0.33 [0.23-0.47]* |
| Total |  |
| †Adjusted for cohort effect; sex; country of origin; childhood socio-economic position; unemployment at age 23 and 24; social benefit and sick leave/disability pension at age 23 or 24; marital status, population density, education, history of psychiatric disorder and history of suicide attempt at age 24  ‡Reference  *P<0.001 | |
